# Private sector quality interventions to improve maternal and newborn health in low- and middle-income countries: a scoping review

**DOI:** 10.3389/fpubh.2025.1332612

**Published:** 2025-04-09

**Authors:** Anne-Sophie Jung, Nuhu Yaqub, Samantha R. Lattof, Joe Strong, Blerta Maliqi

**Affiliations:** ^1^School of Politics and International Studies, University of Leeds, Leeds, United Kingdom; ^2^Department of Maternal, Newborn, Child, Adolescent Health and Ageing, World Health Organization, Geneva, Switzerland; ^3^School of Politics and International Relations, Queen Mary University London, London, United Kingdom

**Keywords:** maternal health, newborn health, private sector, WHO NQPS, quality of healthcare, healthcare delivery, healthcare service delivery, quality interventions

## Abstract

**Introduction:**

The private health sector provides significant maternal and newborn health (MNH) services in mixed healthcare systems in many low- and middle-income countries (LMICs), making it an essential partner in achieving universal health coverage (UHC). Although some studies have mapped the private sector’s activities in MNH care in LMICs, limited knowledge exists about specific quality of care interventions. This scoping review addresses this gap by mapping quality of care interventions implemented by private healthcare providers for MNH care in LMICs.

**Methods:**

Following the Preferred Reporting Items for Systematic Reviews and Meta-Analyses extension for Scoping Reviews (PRISMA-ScR), nine electronic databases were searched. Studies were included if they examined an intervention primarily designed to deliver MNH care by the private sector in LMICs.

**Results:**

A total of 11,922 titles and abstracts were screened, with 38 meeting the inclusion criteria. Qualitative and quantitative data were extracted for descriptive statistics and thematic analysis, focusing on maternal mortality, maternal morbidity, newborn mortality, newborn morbidity, quality of care, experience of care, private sector care usage, and stillbirth. Findings are presented as a systematic narrative synthesis using the WHO’s National Quality Policy and Strategy (NQPS) framework’s quality intervention groups. While various interventions exist, public–private partnership (PPP) schemes and vertical programmes have received more scholarly attention. Most studies emphasised health outcome indicators.

**Discussion:**

We argue that outcome reporting should be diversified to include stakeholders’ perspectives, helping researchers and policymakers understand how governments can engage the private sector in sustainable partnerships that strengthen health systems and advance UHC with quality. Interventions should be people-centred, incorporating feedback mechanisms that promote accountability and empower intended beneficiaries.

## Introduction

Private sector providers play a crucial role in delivering health services in low- and middle-income countries (LMICs) (1–3). The private sector is present in the majority of healthcare systems and contributes significantly to providing maternal and newborn healthcare (MNH) services (3). Analysing 70 LMICs, Grépin (4) found that the private sector provides over one-third of maternal health services. Campbell et al. show that in LMICs, the private health sector provides on average 44% of antenatal care (ANC) and 40% of childbirth delivery services (5). As such, private sector providers are essential to achieving UHC in mixed (public–private) healthcare systems (6). It is therefore important to broaden the evidence base to better understand the role private providers play and could play, to facilitate more effective engagement, and to routinely integrate them in the improvement of MNH care delivery (6).

Despite the involvement of the private sector in MNH care delivery, few existing studies systematically examine the extent of the private sector in the delivery of MNH care across LMICs (7).

This scoping review aims to answer the question: What quality interventions have been designed and implemented by private health sector providers to deliver quality MNH care? We define private sector healthcare providers as individuals and organisations delivering health services that are not owned or directly controlled by governments, such as for-profit and not-for-profit entities, including private for-profit healthcare providers, charities, philanthropic organisations, faith-based organisations, and non-governmental organisations (NGOs) (8). Traditional and informal private sector providers are beyond the scope of this review, as are private sector providers that support the delivery of health services (e.g., supply chain, education, training, and insurance providers) (9). Maternal health covers the wellbeing of women throughout pregnancy, childbirth, and the postpartum phase while perinatal health covers the period from 22 completed weeks of gestation to 7 days after birth. Newborn health focuses on the first month of a baby’s life (10).

## Methods

We conducted a transparent and reproducible scoping review using the Preferred Reporting Items for Systematic Reviews and Meta-Analyses extension for Scoping Reviews (PRISMA-ScR) tool and reporting guidelines for protocols (11, 12). We chose a scoping review to identify and document private-sector MNH interventions in LMICs, anticipating varied types of evidence. The searches, application of in/exclusion criteria, screening, and data extraction were conducted following a rigorous protocol and utilising data extraction tools based on the PICOTS (Patient population, Intervention, Comparator, Outcome, Timing, and Setting) criteria (Table 1).

**Table 1:** PICOTS criteria used in the scoping review.

PICOTS	
Populations	Women during pregnancy, childbirth, and postpartum; and newborns
Interventions	An implemented intervention that is primarily and explicitly designed to deliver maternal and newborn health services by the formal private health sector
Control	Not necessary
Outcomes	Quantitative, qualitative, or mixed-methods data on: maternal morbidity maternal mortality newborn morbidity newborn mortality one of the six components of quality care (i.e., safety, effectiveness, timeliness, efficiency, equity, and people-centred care) experience of care, including respectful care use of formal private-sector care during pregnancy, childbirth, and postpartum stillbirth
Timeframe	1 January 2002 to 1 June 2021
Setting	Low- and middle-income countries

The scoping review considered peer-reviewed and non-reviewed studies, papers and conference abstracts, presentations, and reports on private sector involvement in MNH care interventions in LMICs classified according to the World Bank Atlas, published between 01 January 2002 and 01 June 2021. Studies had to report qualitative and/or quantitative data on one of the following outcomes: maternal mortality; maternal morbidity; newborn mortality; newborn morbidity; quality of care (QoC); experience of care, including respectful care; the use of private sector care during pregnancy, childbirth, and postpartum; and stillbirth.

Nine electronic databases were searched using combinations of relevant search terms (Table 2) adapted to each database. The searched databases are the MET, HCPPR, Cumulative Index to Nursing and Allied Health, Excerpta Medica Database, International Bibliography of the Social Sciences, PubMed, ScienceDirect, Web of Science, and WHO Institutional Repository for Information Sharing. We included items in English, French, German, and Italian. We followed the PRISMA-ScR flow approach using our published protocol (9).

**Table 2:** Search terms and their combinations.

1. Private health sector terms	2. Intervention or study type terms	3. Population terms
private sector	arrangement*	*Antepartum terms*
for-profit	evaluat*	antenatal
for profit	initiative*	antepartum
public-private	intervention*	pregnan*
private enterprise*	model*	prenatal
NGO	package*	trimester
non-government*	pilot*	*Intrapartum terms*
self-financ*	program*	birth*
charit*	project*	childbirth
faith-based	provision*	intrapartum
private health sector	regime*	matern*
mixed health system*	scheme*	obstetric*
integrated health system*	strateg*	parturition
non-state	trial*	partus
non-profit		perinatal
not-for-profit		stillbirth*
		*Postpartum terms*
		mother*
		newborn*
		neonat*
		postnatal
		postpartum
		puerper*

*means that the search will include all variations of the word that start with the root before the asterisk.

For inclusion, studies must evaluate service delivery interventions that have been primarily and explicitly designed to deliver MNH care by the formal private health sector in LMICs. As indicated in the PICOTS criteria (Table 1), included studies must report quantitative, qualitative, or mixed-methods data on at least one of the following outcomes: maternal morbidity, maternal mortality, newborn morbidity, newborn mortality, components of quality care (e.g., safety, effectiveness, timeliness, efficiency, equity, and people-centred care), experience of care (including respectful care), use of formal private sector care during pregnancy, childbirth, and postpartum, or stillbirth. For inclusion, items must be research articles, reports, or descriptions of the implemented services/interventions. As we are focused on service delivery, we limited the private health sector to formal private providers who deliver direct medical care (e.g., private health facilities, private healthcare providers, civil society organisations delivering MNH care, and charities delivering MNH care). Since MNH needs may be met through primary healthcare, titles and abstracts that mentioned primary healthcare without specific mention of MNH care were moved forward to full-text screening for verification of the population and intervention.

Items were excluded if they reported aggregated service delivery data (e.g., combined outcome data from public and private health sectors). The private non-health sector (e.g., private cars or buses transporting pregnant women to health facilities) and private sector entities that do not deliver direct medical care were excluded. For example, we excluded private pharmaceutical providers (including pharmacies) and private health insurance companies. We also excluded study protocols.

As a scoping review, we did not assess the risk of bias in individual studies. We analysed and synthesised the private sector involvement in MNH care delivery in LMICs by six themes with 103 indicators. These themes include descriptive statistics, intervention background, intervention details, outcomes, evaluation, and study description. One researcher (ASJ) coded all studies, and 10 randomly selected studies were analysed by another researcher (SRL) for quality control. In cases where outcomes deviated, clarification was sought through discussion between the researchers. We decided to include more information in cases of doubt and repeated this process until we reached a consensus.

**Figure 1:**
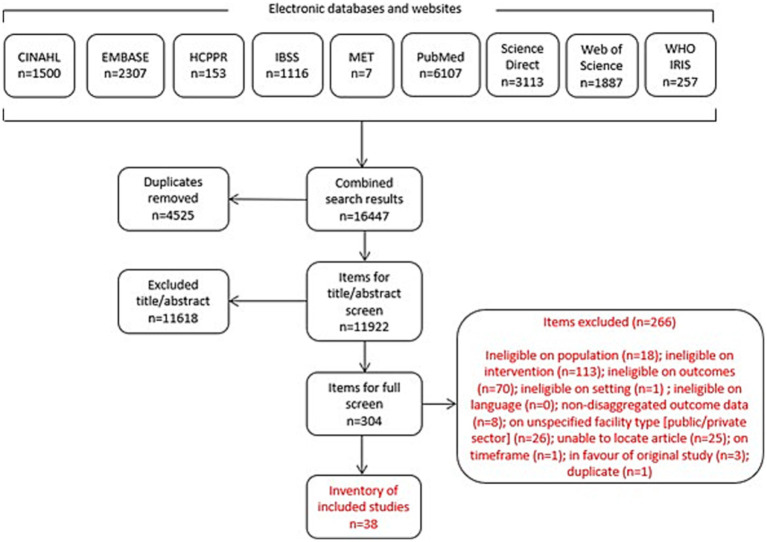
Screening results.

Our search generated 16,447 items for screening (Figure 1). After removing duplicates, the remaining 11,922 items were screened by title and abstract (TIAB) for inclusion. We determined the eligibility of all items, and unclear cases were discussed. In cases where exclusion could not be determined based on TIAB, the full text was reviewed. After TIAB screening, 304 studies remained for full-text review. Decisions were made in favour of an inclusive approach when questions arose; ultimately, 38 studies met all inclusion criteria.

We present the data using a systematic narrative synthesis, organised according to the QoC intervention groups proposed by the WHO’s National Quality Policy and Strategy (NQPS) guidance (13). Thematic reports are supplemented by tables of descriptive statistics on included studies and their outcomes. We excluded studies on private sector entities not delivering direct healthcare (e.g., teach-the-teacher, transportation services), as they do not solely focus on community and stakeholder engagement.

## Framework

In this scoping review, we use the four groups of quality interventions as outlined by the WHO’s NQPS framework to structure our analysis (13). The NQPS framework provides a practical approach for developing policies and strategies to improve the QoC. Its four quality-related interventions are: system environment; reducing harm; improvement in clinical care; and patient, family, and community engagement and empowerment (Table 3).

**Table 3:** Illustrative quality interventions according to the WHO’s Handbook for National Quality Policy and Strategy, 2018.

Intervention	Definition
System environment	Registration and licensing External evaluation and accreditation Clinical governance Public reporting and comparative benchmarking Performance-based financing and contracting Training and supervision of the workforce Medicine regulation
Reducing harm	Inspection of institutions for minimum safety standards Safety protocols Safety checklist Adverse event reporting
Improvement in clinical care	Clinical decision support tools Clinical standards, pathways, and protocols Clinical auditing and feedback Morbidity and mortality reviews Collaboration and team-based improvement cycles
Patient, family, and community engagement and empowerment	Formalised community engagement and empowerment Health literary Shared decision-making Peer support and expert patient groups Patient experience of care Patient self-management tools

## Results

We first present the descriptive statistics for studies included in this scoping review, followed by findings categorised by the four groups of the NQPS interventions. Twenty-two articles (58%) studied system environment interventions (group 1), two focused (5%) on harm reduction (group 2), nine (24%) addressed improvements in clinical care (group 3), one (3%) analysed patient, family, and community engagement and empowerment (group 4), and four (11%) described multi-component interventions. Quality-related interventions are often interrelated, with many frequently covering multiple components. For this scoping review, we categorised interventions based on the aspect most emphasised by the authors, in alignment with the NQPS quality-related intervention groups.

### Descriptive results

We reviewed 38 articles, which examined a total of 40 interventions. The majority of studies focused on interventions in South Asia (72.5%), with 45% specifically examining interventions in India, followed by 17.5% in Bangladesh (Table 4). Fifteen percent of interventions were in Sub-Saharan Africa. Studies on interventions in the Middle East, North Africa, and Latin America were notably absent. Table 4 provides an overview of interventions by region and country according to the World Bank Atlas classification. Less than a quarter of the studies (23.7%) were published by first authors affiliated with high-income country institutions, while nearly one-third originated from first authors based in India (31.6%).

**Table 4:** Included interventions by region and country.

Region/Country	Number of studies included in final inventory	in %
East Asia and Pacific: *Cambodia, Indonesia, Malaysia, and Myanmar*	4	10
Europe and Central Asia	0	0
Latin America and the Caribbean: *Guatemala*	1	2.5
Middle East and North Africa	0	0
South Asia: *Bangladesh (7) and India (18)*	29	72.5
Sub-Saharan Africa: *Benin, Kenya, Nigeria, Uganda, and Zambia*	6	15
Total	40	100%

Eighty-nine point five and percent of studies analysed interventions in lower-middle-income countries, 2.6% focused on low-income countries, and 5.3% on upper-middle-income countries. One study covered multiple income levels. The majority of studies were quantitative, with 89.5% (*n* = 34) relying exclusively on quantitative data. Four studies included either qualitative data (*n* = 1) or both quantitative and qualitative data (*n* = 3). Half of the studies focused on the supply side, and 42.11% covered both the supply and demand side. Only 5.26% (*n* = 2) focused solely on the demand side. Table 5 provides an overview of study characteristics, including publication type, country income group, and data collection methods.

**Table 5:** Characteristics of studies included.

Characteristic	Number of studies included in final inventory	%
Type of publication		
Peer-reviewed journal article	26	68.4
Non-peer-reviewed journal article	1	2.6
Report	2	5.3
Book/book chapter	–	–
Other	9	23.7
Country income group		
Low	1	2.6
Lower-middle	34	89.5
Upper-middle	2	5.3
Multiple	1	2.6
Intervention evaluated		
Yes	35	92.11
No	3	7.89
Type of evaluation		
Impact	27	71.05
Process	3	7.89
Impact and process	2	5.26
Multiple	1	2.63
Economic	1	2.63
Other	1	2.63
Unclear/not specified	3–	7.89
Study type		
Randomised controlled trial	2	5.3
Controlled clinical trial	–	–
Cohort analytic	1	2.6
Case–control	2	5.3
Cohort (before and after)	3	7.9
Interrupted time series	–	–
Qualitative	2	5.3
Mixed methods	4	10.5
Regression analysis	12	31.6
Other	8	21.1
Unclear/not specified	4	10.5
Control or comparator group		
Yes	13	34.21
No	25	65.79
Unclear/not specified	–	–
Type of data		
Quantitative	34	89.5
Qualitative	1	2.6
Both	3	7.9
Longitudinal data		
Yes	7	18.4
No	28	73.7
Unclear/not specified	3	7.9

Just under one-third of studies (26.3%) involved multiple study populations, with 18.4% focusing on healthcare providers or women during childbirth, respectively. The majority of studies were conducted at the sub-national level (57.9%) or health facility level (23.7%) (see Table 6 for the study population and geographical scope).

**Table 6:** Geographical level and study population of studies included.

Geographical level	Number of studies included in final inventory (%)	Study population	Number of studies in final inventory (%)
National	1 (2.6)	Pregnant women	2 (5.3)
Sub-national	22 (57.9)	Women during childbirth	7 (18.4)
Local	4 (10.5)	Postpartum mothers	1 (2.6)
Health facility	9 (23.7)	Newborns	3 (7.9)
Global	0 (0.0)	Healthcare providers	7 (18.4)
Multiple levels	2 (5.3)	Multiple answers	10 (26.3)
		Other	6 (15.8)
		Not applicable	2 (5.3)
Total	38 (100.0)		38 (100.0)

### System environment

Twenty-three out of the thirty-eight articles focused on system environment quality-related interventions. The primary interventions studied were performance-based financing and contracting (18 studies), where payments to healthcare providers were based on specific performance measures to improve quality. Payments were typically part of the total compensation and could be determined using various financing methods (13). The majority of these studies (*n* = 18) focused on public-private partnerships (PPPs), aimed at contracting in healthcare providers or contracting out to private sector providers. One study examined franchise models.

Nearly 50% of studies (*n* = 11) focused on two PPPs: the Chiranjeevi Yojana (CY) scheme in Gujarat, India (14–23), and its counterpart, the Janani Suraksha Yojana (JSY) in Maharashtra (24, 25). In these studies, private sector involvement involved public funding for private obstetricians to provide free health services to poorer populations. All reviewed studies reported positive outcomes, such as increased maternal mortality reduction, healthy deliveries, improved access to care, choice of delivery location, number of providers trained, increased number of ANC check-ups, and vaccination rates. However, it remains unclear whether this increase occurred primarily among the targeted populations (e.g., women below the poverty line) or broader lower-income families.

Four studies analysed contracting models beyond the two named PPPs. Two studies examined contracting out health services to NGOs in Pakistan (26) and Bangladesh (27), respectively. Zaidi et al. (26) found higher attendance by literate patients and increased out-of-pocket spending for ante- and postnatal services. Mercer et al. observed a decline in mortality in NGO-contracted areas (27). Caplain et al. analysed a contracting model, which provided a new degree to young healthcare providers to qualify as independent private community general practitioners (GPs) in rural areas (28). Cristia et al. (29) evaluated a large contracting programme in Guatemala in 1996, noting that better results were achieved under contracting-in models, with a 24% increase in nurse and physician utilisation for prenatal care.

One study analysed three franchise models designed to improve access to and the quality of maternal care in the private sector, while also better understanding the socio-economic profile of clients (30). The franchise covered only some of the services, making it a ‘fractional franchise’ model. Haemmerli et al. (30) observed that despite focusing on serving poorer populations, users of these services were primarily from higher wealth quintiles.

Three studies examined the impact of training and mentoring healthcare staff (31–33). Baig and Shahid, for example, analysed a competency-based training programme in Pakistan targeting community midwives and health facilities (32). This programme consisted of group- and classroom-based training of service providers, hands-on practice on mannequins and patients in clinical settings, and the development of standardised learning resource packages (32). The intervention targeted 112 community midwife-led clinics (CMW), 93 public (Department of Health) facilities, 109 private facilities, and 327 public–private partnership-led facilities across 15 districts in Sindh province. According to the authors, the preliminary analysis found significant improvements in the quality of antenatal, childbirth, and postnatal care at community midwife-led centres that received training, compared to those that did not (32). Two additional studies in Pakistan (31) and Nigeria (33) also reported improvement in the quality and availability of health services provided through the private sector, following the introduction of training programmes for community midwives operating in the private sector. Sini et al. analysed the effectiveness of a mobile obstetrics monitoring programme that used software solutions to better connect patients in rural areas with midwives and physicians, serving as a model for community-based antenatal care delivery in Indonesia (34). The study demonstrated that the programme improved decision-making and enhanced the skills and knowledge of the midwives.

### Reducing harm

Two studies examined interventions aimed at reducing harm. Baniamin et al. analysed overtreatment by physicians in Bangladesh, revealing a higher rate of caesarean deliveries in private clinics than in public or non-profit facilities, suggesting a potential issue with overtreatment (35). The findings suggest that introducing competition in healthcare services may not effectively reduce unnecessary medical procedures, as increased competition does not necessarily lead to decreased overtreatment. Day et al. described the challenges and successes of implementing a Perinatal and Maternal Death Audit in a rural hospital in Bangladesh (36), emphasising the value of such audits and the importance of emotional support for healthcare workers to prevent emotional fatigue.

### Improvement in clinical care

Nine studies focused on improving clinical care. Three studies examined morbidity and mortality reviews as a tool for clinicians to engage in self-reflection, identify areas for improvement, and promote a culture of learning (27, 37, 38). The remaining studies (*n* = 5) described and/or evaluated clinical programmes addressing access, auditing, and clinical standards (39–44). For example, Bhartia et al. (45) examined a programme that reduced caesarean section rates in a private, non-for-profit Indian hospital through a combination of hiring more nurses and experienced physicians, implementing audits, regularly presenting and reviewing data, developing clinical guidelines, establishing shared decision-making structures, and collaborating with other healthcare facilities. Jolly et al. (39) analysed a similar programme addressing maternal, neonatal, and child health issues among slum dwellers in Bangladesh. The study focused on clinical indicators, standards, and morbidity and mortality rates, and clinical audits to improve care. Similarly, Dangoria et al. (44) described access to ANC through non-profit hospitals in India, while Imtiaz et al. (40) measured the uptake of maternal and child health services after implementing a PPP in Pakistan.

### Patient, family and community engagement, and empowerment

Interventions in this group sought to formalise community engagement and empowerment, enhance health literacy and shared decision-making, link people with shared experiences through peer support and expert patient groups, enhance patient self-management tools, and incorporate patient experience of care to design improvements in clinical care. Gatakaa et al. studied Ubuntu-Afya Kiosks in Kenya, a network of community-run medical centres developed through a private-community-government partnership. The study demonstrated improved uptake of skilled delivery and ANC, with the partnership model helping to create a self-sustaining healthcare service (46).

### Multi-component interventions

Quality interventions are often interrelated. Four studies addressed multiple components. For example, Yadav et al. evaluated a private sector quality improvement programme in India that involved training healthcare workers, in-facility mentoring, advocacy for prioritising resources, data recording and reporting, peer assessments, and combining system environment and clinical care improvements (47). Hossain (48) and Baqui et al. (49) both analysed interventions aimed at improving the system’s environment. These included, for example, training private-community skilled birth attendants, formalising community engagement and empowerment through ‘social entrepreneurship capacity building’ (48), and conducting home visits by community health workers (49). Other studies, such as those related to the ‘Saving Mothers, Giving Life’ initiative in Uganda and Zambia, focused on enhancing integrated service delivery by linking public and private inputs, institutionalisation of maternal mortality surveillance, and auditing at the district level (50).

## Discussion

This scoping review sought to answer the question of what service delivery interventions have been designed and implemented by private health sector providers to deliver quality MNH care. Using the WHO’s NQPS framework, this review demonstrated the breadth of private sector involvement in MNH care delivery.

The review shows that much of the private sector involvement focuses on system environments, particularly through performance-based financing and service contracting via PPP models. Nearly half of the studies in this review focused on PPPs, primarily in India. While these provide important insights, the engagement of policymakers with the private sector—especially in governance and service provision—appears skewed towards PPP models, which is also reflected in academic research and published articles. The majority of studies monitored access to quality care delivered by the PPPs, sparking ongoing debates about whether quality care can be better delivered through these sector providers and whether the models actually reach the targeted populations. This review has also revealed the prominence of interventions focused on training healthcare workers.

While increasing access to MNH care and improving provider training are key concerns for policymakers, it is critical that these interventions do not exacerbate underlying structural inequities. The reviewed studies engaged less with the structural factors and inequalities that impact the long-term success of MNH interventions. These factors include proper targeting, community and stakeholder involvement in designing the interventions, and linkages to ongoing health reforms that can improve sustainability. This is particularly important as many initiatives lose momentum when donor support ends or the project concludes. Another significant research gap involves analysing feedback mechanisms and considering the experiences of those receiving the interventions. Healthcare delivery must be people-centred, incorporating feedback and ensuring accountability at all levels. The following discussion will elaborate on these points, suggesting avenues for future exploration.

### Context and sustainability

Health systems are deeply embedded in social, political, and economic contexts. As the private sector involvement in LMICs grows, concerns about equity, health financing, and access persist. However, no study specifically analysed interventions in relation to contextual factors or examined how structural drivers and political or economic contexts influence implementation and outcomes. While several studies targeted low-income households, they primarily measured whether the target groups were reached. An exception is Chaturvedi and Randive’s (25) study of the JSY PPP scheme in Maharashtra, which identified key design implementation issues, including specificities of what the scheme covers or neglects to cover.

Similarly, few studies analysed organisational factors. Understanding how interventions are designed, who decides on them, and how they are managed is critical to evaluating their success or failure.

The lack of focus on sustainability was also evident, as most interventions were project-based and dependent on donor funding. Few studies addressed how an intervention could lead to long-term change or become a regular part of health service provision.

Implementation science could offer a useful framework for further investigating private sector involvement in MNH care quality improvement. By focusing on sustainability, scalability, and the contextual factors (both inner and outer) that shape intervention delivery (51), implementation science frameworks (e.g., CFIR, RE-AIM, and the Health Equity Implementation Framework) can guide researchers’ process evaluations of interventions aimed at improving the quality of MNH care delivery (52–54).

### People-centredness and accountability

With only three studies focusing on harm reduction and one on individual, family, and community engagement and empowerment, this review highlights a significant gap in the literature. The emphasis on patient-level health outcome indicators diverts attention from understanding patients’ experiences of care. Healthcare should be people-centred, grounded in human rights principles, and incorporate feedback from those it serves (55). Establishing feedback structures where communities can engage in intervention development, implementation, and evaluation is essential. These structures can also contribute to accountability at various levels.

Few studies incorporated stakeholder views—whether from physicians, nurses, or patients—and even fewer analysed mechanisms of accountability. Only seven studies reported on the experience of care, often through patient satisfaction surveys. Yet, for sustainable service delivery and improved MNH care quality, interventions should be co-produced with the communities they aim to serve (56). This approach is vital in addressing gendered, racialised, and other structural inequalities. Co-producing knowledge with communities has been shown to create feasible and acceptable solutions to healthcare concerns (56). Researchers and policymakers should diversify research designs, methods, and indicators, incorporating PROMS and PREMS (patient-reported outcome and experience measures) to enhance the QoC. However, these tools should be adapted to the specific contexts of LMICs.

### Strengths and limitations

In line with the scoping review’s aim of providing a broad overview, this review included a variety of literature, from peer-reviewed research papers to conference abstracts. The strengths of the scoping review lie in its wide-ranging synthesis of private sector MNH care delivery in LMICs, identifying gaps and further research areas. The findings are directly applicable to practice and research in this field. However, there are limitations. Scoping reviews have inherent limitations because the focus is to provide breadth rather than depth of information on a particular topic. Our search focused on outcomes more easily measurable in quantitative terms, which may have resulted in a bias towards quantitative indicators over qualitative ones. We conducted the search in English only, which limited the included studies to those disseminated in English. As such, our results are generalisable to scoping reviews written in English. Additionally, the geographical focus of the studies was limited, with a bias towards South Asian countries, in particular India and its PPP models. Additionally, as much of the literature identified during this review focuses on PPPs, this review may not appeal to those interested in private sector impact. We also acknowledge that since July 2021, more studies will inevitably be published, yielding further insights into private-sector involvement in MNH care interventions. In the future, re-running the search will be valuable to update and refine the findings. Given these limitations, caution is advised when interpreting the findings.

## Conclusion

This scoping review sought to identify the type of private-sector MNH care interventions in LMICs. By reviewing 38 studies through the NQPS’ quality interventions framework, the review highlights the wide-ranging involvement of the private sector in MNH care in LMICs. Private sector interventions show potential impact in reducing harm, improving front-line healthcare services, and building systemwide capacity for quality improvement. As the majority of studies prioritise health outcomes over patient experience, stakeholder perspectives, and feedback mechanisms, potential gaps arise, underscoring the need for more research to improve care quality in the private sector. The private sector’s role in MNH care is diverse and requires systematic research to leverage its contributions effectively. This review underscores the need to focus on how private sector MNH quality interventions are designed, by whom, and how knowledge is co-produced with those receiving care. Sustained funding and long-term planning are essential to ensuring these interventions contribute to the broader goal of strengthening mixed healthcare systems and achieving UHC.

## Data Availability

The original contributions presented in the study are included in the article/Supplementary material, further inquiries can be directed to the corresponding author.
